# Hospitals of Paris

**Published:** 1858-01

**Authors:** Richard J. Dunglison

**Affiliations:** Philadelphia


ART. XI.—*Hospitals of Paris.* Extracts from letters of RICHARD J. DUNGLISON, M.D., of Philadelphia.

* * * * * * OF the Medical Schools and Hospitals of Paris you know much from the report of those who have been able to partake of their advantages. And yet, if you have formed any idea of their superiority over our own, you will find, upon a more intimate knowledge of their merits from personal inspection, that there are many points in which they fall far short of ours.

In the first place, as regards the lecture-rooms, they are by no means as imposing as you would imagine the annual influx of medical visitors in attendance upon the different teachers ought to have the effect of making them. Compared with the lecture-rooms of our medical colleges in America, they are insignificant. I was at Hôtel-Dieu yesterday morning, and found it well filled with students, but in a room not very comfortable, and not well adapted for their convenience. The subject of ventilation has not been studied very closely in the construction of either the hospitals or lecture-rooms. The entrances to the latter are, as a general rule, below the level of the room, and there is scarcely ever more than one small door and a narrow staircase leading into the auditorium. There are few windows, and these, I notice, are generally kept closed; the entrance at the foot of the stairs and the steps themselves are almost always blocked up with students: no draught of air can possibly be maintained under these circumstances, and the young men are expected to enjoy the hour or hour and a half they spend there, at a temperature at which fans and fresh air would be a luxury. M. Ricord's plan of lecturing under the trees is the most agreeable; his lecture-room in the open air is the best ventilated one in Paris.

As regards the study of specialties, for which the French capital has acquired an extensive reputation, there are many who maintain that Paris alone possesses pre-eminent advantages of this kind. Overlooking the equally important and perhaps more ample hospital resources of Vienna, they cannot cast their eyes beyond Paris, or are so much dazzled by what is presented to them in that city, that they are blinded to the true condition of medical science elsewhere. If the observation proceeds from one who is not an American he does not look beyond the Atlantic, or he might, perhaps, in our own city of Philadelphia, find valuable means of private instruction offered to the medical student, both during an attendance upon the winter courses of lectures, and in special courses upon important branches in the intervals between the winter sessions. But it has been the fashion to exalt Paris into a sort of medical Mecca, to which trans-Atlantic and cis-Atlantic pilgrimages are to be made; and it is likely enough that this reputation will exist for some time, however undeserved it may be, or whatever position other cities may occupy in the scale of medical merit.

Of lectures and lecturers I have something to say, although I have scarcely been long enough here to give you anything but first impressions. I am confident, however, that they would be confirmed by further experience, and they are the unbiassed convictions of one who is not accustomed to judge over-harshly; so that you can receive them as at least honest criticisms. I determined, immediately after my arrival here, to devote a few hours of each morning to visiting hospitals, and on Thursday morning last commenced with the Hôpital du Midi, where M. Ricord lectures and holds his clinics.

This hospital was founded toward the close of the last century, upon the site of the old convent of the Capuchins. It has an ancient look even in its modern state, and has, I believe, about six hundred beds. M. Ricord went through his wards prescribing for the benefit of his patients, and joking for the amusement of his pupils. He then lectured for about an hour and a half under the trees upon sarcocele, varicocele, etc. But I derived less profit from the lecture than satisfaction at seeing, for the first time, one whose labors in the investigation of the nature and treatment of a special class of disorders has made him so widely known. He talks so fast and puns so incessantly, that it is very difficult for a foreigner to follow him; and they who are the most familiar with the language in all its idiomatic observances find it at times quite a task to appreciate the points, when his countenance evidently betrays that he has perpetrated what he esteems to be excellent jokes. I enjoyed the lecture, however, from its peculiarities and its novelty.

We had afterward a clinic in the open air, and several operations for phimosis and paraphimosis, of which I could see but little. There, as elsewhere, when you are most anxious to observe what is going on, the students crowd around the bed, hang over the patient, and, without any regard to each other's personal comfort, place themselves in such sort that many must be obliged to give up all idea of seeing the operation. You find yourself tightly wedged in a solid mass, unable to extricate yourself, and without power to elevate yourself so as to look over heads, or to depress yourself to see under axillæ. I was able, however, to catch a glimpse occasionally of one case, which, when placed under the influence of chloroform, very nearly proved fatal from its effects. I have never seen any one resuscitated who seemed so near death. Fifteen or twenty minutes elapsed before he gave any symptoms of returning animation, and then only after he had been well beaten by all the *externes* and *internes*, and artificial respiration by insufflation, etc., had been thoroughly practiced. One patient, who bore an operation remarkably well without being chloroformized, was told by M. Ricord that he was evidently born for that *spécialité*,—viz. to be operated on. The near approach to death of the subject of his operation seemed to disturb his equanimity but little, and he punned away and preserved as comical an expression of countenance as if nothing unusual was occurring.

On Friday, accompanied by a young American professional friend—Dr P. of Philadelphia—I visited *La Charité* and the *Hôpital Militaire du Val-de-Grâce*. At the former I met M. Velpeau; went through the wards with him, and saw him perform one or two little operations, such as opening abscesses of an unimportant character. I also heard him lecture upon surgical affections of the inferior maxillary bone, but was compelled to leave before I was able to glean much useful information. The class here, as at most of the hospitals, is small; M. Trousseau, I am told, has the largest attendance. M. Ricord's class, on Thursday, was much more numerous than M. Velpeau's on Friday. The latter teacher is becoming advanced in years; he goes from bed to bed without saying much, and what he does say is not very audible to those around the patient. As a lecturer he is not remarkable: he lives upon his former popularity as a surgeon, but possesses no very strong claims to attraction as a clinical teacher, at the present time. Such, at least, was my conviction,—a mistaken idea, perhaps, formed at first sight, which after-experience might correct. La Charité has accommodation for fewer patients than Hôpital du Midi. It contains beds for four hundred, and is intended, I believe, for the reception of some of the rarer forms of disease. It was built in 1613, for the use of the Frères de la Charité.

After leaving M. Velpeau's lecture-room, I went to the Val-de-Grâce. Baron Larrey, to whom I had a letter of introduction from my father, kindly put me under the charge of one of his officials, and I was thus enabled to see the large establishment to great advantage. The Museum is a very interesting one, and contains many curious pathological specimens, some of them deposited by the present Baron, others profoundly interesting as evidences of the skill of his celebrated father. Nearly two and a half centuries ago this hospital was built, to be devoted to religious purposes, as a convent, under the patronage of Anne of Austria. It is now the great military hospital of Paris, and has accommodations for about 1500 patients.

Yesterday I had the pleasure of hearing M. Trousseau lecture on vaccination at Hôtel-Dieu. The class was larger than any I had seen; about two hundred or two hundred and fifty students were present, many of them Americans, and some of them English. M. Trousseau is a capital lecturer; so clear that it is not at all difficult for a foreigner to follow him, and on this account, as well as for other substantial reasons, he is, probably, more popular, as a teacher, than any of his professional brethren. His manner, too, is that of a gentleman, and his voice pleasant to listen to. Hôtel-Dieu is probably the most ancient hospital in Europe, being now at least 1200 years old. Different rulers have taken it under their special protection, and afforded it substantial pecuniary aid; while rich legacies have been bestowed upon it by wealthy citizens of Paris. The establishment is divided by an arm of the river, but the two portions are connected by a private bridge. Twenty-three rooms are devoted to the purposes of the hospital, and contain 1200 beds. There are certain classes of diseases, which the regulations of Hôtel-Dieu prohibit from being received, such as those of children, and obstetrical, incurable, insane, and venereal cases, as well as chronic affections. This rule prevents the hospital from being over-crowded.

After leaving Hôtel-Dieu, I visited *La Pitié*, but, unfortunately, arrived there too late for M. Maisonneuve's lecture. He was talking to a few young men on the subject of *Écrasement* and the *Écraseur*, and had several instruments lying near him,—some of them like those which M. Chassaignac is in the habit of using; others, M. Maisonneuve's modification of the original: all of them rather formidable instruments to a novice in the profession. MM. Chassaignac and Maisonneuve are the great *Écraseurs* of Paris; the former, perhaps, makes it more of a hobby, but both are extremely fond of employing this method of surgical relief wherever practicable. The former never neglects an opportunity of using it, but I do not think he renders himself more popular, from this constant employment of his own invention, than others, who, with their patient's interest always in view, vary their treatment according to the nature of the case, instead of experimenting with the idea of making all cases respond to one treatment.

This morning I visited *Lariboissière*, the most recently constructed hospital in Paris. Commenced in the year 1846, it has had, in the short space of time which has elapsed from that event to its present completed condition, the unusual fortune to be known at different times under three distinct names. Founded at a time when Louis Philippe was popular with the people of Paris, it received the name of *Hôpital de Louis Philippe*, and that title it retained until the capricious tastes of the French people changed the form of national government and rendered them miserable, for a while, under the anarchy of a Republic swayed by the whims of the populace. It was then known as the *Hôpital de la République*, but the existence of this title was almost as short-lived as the form of government that gave it its name. The spirit of change again invaded the French people, and when the Empire succeeded to the Republic, the name attached to this hospital became, of course, odious in the sight of the imperial authorities, as well as of the imperially disposed Parisians. It was then that, in compliment to the Countess Lariboissière, who had handsomely bequeathed eight millions of francs to the use and purposes of the hospital, it became known under the name which it now holds, and which it will probably retain permanently.

Here, in former times, was situated the Convent of the Lazarists and all its vast estates. The ground is now partly occupied by the most complete and best-ventilated establishment of the kind in Paris. What has been neglected in other hospitals, as regards architectural beauties and general details, tending to the comfort and restoration of their occupants, has here received every attention; and the attachés of Lariboissière may congratulate themselves upon the satisfactory manner in which everything has been arranged. M. Chassaignac, whose name has been heard much more frequently of late years, in consequence of the attention which his *Écraseur* has excited, lectured here to a moderate class. He is a tolerable lecturer, and has the good fortune to have an amphitheatre to receive his students in, which, although small, is comfortable compared with those of the other hospitals. Great room for improvement, however, still remains, and one cannot but wonder why the continual influx of large numbers of young medical men into Paris should not, ere this, have had the effect of attracting the attention of those in authority at the hospitals to the amelioration of this important matter. The Lariboissière lecture-room is the first step toward a change.

I have been so short a time in Paris that I have not been able to make myself acquainted with any but the prominent medical attractions. A residence of some duration is requisite to become intimately conversant with the routine of the hospitals, the peculiarities of lecturers, and modes of instruction. The French capital abounds in establishments for the relief of the sick and suffering, and it is a matter of historical interest to compare their present superior condition with the deplorable state of wretchedness, and the consequent mortality, which prevailed up to the close of the last century in these ill-conducted charities. It was not until investigations were made, with a view to improvement, that the people of Paris became fully aware of the almost incredible neglect into which they had fallen. New plans were devised for ameliorating them, and, under a new system of management, they speedily assumed their present advanced condition.

